# Evaluation of the Lower Trapezius Muscle Using Ultrasound Panoramic View (a Novel Approach): An Intra- and Inter-Rater Reliability Study

**DOI:** 10.3390/ijerph17197123

**Published:** 2020-09-29

**Authors:** Samuel Fernández-Carnero, Alejandro Garrido-Marín, Alexander Achalandabaso-Ochoa, Alejandro Ferragut-Garcías, Rubén Fernández-Matías, Daniel Pecos-Martín, Tomás Gallego-Izquierdo

**Affiliations:** 1 Department of Physiotherapy and Nursing, Alcalá University, 28871 Alcalá de Henares, Spain; samuel.fernandezc@uah.es (S.F.-C.); daniel.pecos@uah.es (D.P.-M.); tomas.gallego@uah.es (T.G.-I.); 2 CARMASALUD, 28037 Madrid, Spain; alejandro.garrido@carmasalud.com; 3 Department of Health Sciences, Universidad de Jaén, 23071 Jaén, Andalucía, Spain; 4 Department of Nursing and Physiotherapy, Islas Baleares University, 07122 Ciudad de Palma, Spain; alejandro_sft@yahoo.es; 5 Research Institute of Physical Therapy and Pain, Alcalá University, 28871 Alcalá de Henares, Spain; ruben.fernanmat@gmail.com

**Keywords:** lower trapezius, panoramic view, ultrasound, reliability

## Abstract

The panoramic view ultrasound remains uncommon in clinical practice, probably because of its difficulty, high-cost, and lack of research. Morphological changes in muscles have been demonstrated to be related to symptomatology and provide data of interest for clinical assessment. Thus, the aim of this study was to evaluate the measurement reliability of the length of the lower trapezius muscle with the panoramic view ultrasound using a novel tool, SIG_VIP^®^. Twenty healthy volunteers were measured by two expert sonographers using the SIG_VIP^®^ tool with a novel approach. Statistical analyses were performed with the R software. The intraclass correlation coefficient (ICC), standard error of measurement (SEM), minimal detectable change (MDC), and Bland-Altman plots were calculated. All the results indicated good intra-rater reliability (ICC_3,1_, 0.92 to 0.96; SEM, 0.59 to 0.85; MDC, 1.64 to 2.35) and inter-rater reliability (ICC_3,2_, 0.84 to 0.89; SEM, 1.22 to 1.53; MDC, 3.39 to 4.25). The novel system used with the described methodology can reliably measure the length of the inferior fibers of the trapezius muscle. Further research must be conducted to evaluate the reliability in patients and how pathology is related to the length of the lower trapezius muscle.

## 1. Introduction

Ultrasound morphological evaluations were first used for the assessment of muscles as an alternative form of medical diagnosis more than five decades ago [1]. Research began with the aim of obtaining more accurate anthropometric measurements of analyzed muscles [2,3]. These anthropometric measures were correlated with pain and dysfunction in several locations [4,5] to obtain a clinical meaning. Magnetic resonance imaging (MRI) is considered the gold standard in anthropometric measurements. Nevertheless, it is three times more expensive than ultrasound [6] and time consuming. In contrast, ultrasound is more economical, flexible, and has been validated compared to MRI for transversal evaluations demonstrating high correlation versus “gold standard” [7,8,9]. In addition, panoramic ultrasound has shown high degrees of correlation with the ultrasound (US) and MRI with results of 0.94, 0.92, and 0.93.

However, to the best of our knowledge, only two studies have previously used panoramic view ultrasound to evaluate musculoskeletal tissues in the longitudinal axis [10,11] with excellent intraclass correlation coefficients (ICCs) (0.97 and 0.95). A recent study of panoramic ultrasound in the longitudinal axis showed its importance in tissue evaluation [12] because it improves the evaluation of the effects of interventions, affects clinical decisions, and improves the patient experience. Nevertheless, panoramic ultrasound in the longitudinal axis has shown several risks of bias due to the operators’ experience, or the need to move from the screen to the patient’s body as the probe moves long distances.

To decrease these risks of bias, we have designed the SIG_VIP^®^, which almost eliminates the need to deviate the eyes from the screen and allows taking large measurements on long axes, which could not be obtained manually by following a perfect line, as required by a panoramic view. This is new and an advantage thus any great muscle could be studied in long axial. To prove the validity of this concept, we chose to assess the inferior fibers of the trapezius muscle, because when measured by MRI [13] or electromyography, [14] the trapezius fibers show morphological and functional changes compared to healthy subjects. The specific morphology of this muscle requires an approach that evaluates these possible implications for different pathological situations such as shoulder pain. Although the traditional measurement offers a partial view limited to the small size of the ultrasound probe, the panoramic view with a SIG_VIP^®^ approach can screen larger areas.

The aim of the present study was to analyze the reliability of a protocol for sampling the length of the lower trapezius muscle using a customized tool that is being patented [15] and to demonstrate its reliability.

## 2. Materials and Methods 

### 2.1. Study Design and Ethical Approval Statement

An intra- and inter-rater reliability cross-sectional study was conducted according to the Guidelines for Reporting Reliability and Agreement Studies (GRRAS) [16]. Ethical approval was obtained from the Ethical Committee of Alcalá University (CEI/HU/2019/47). Participants gave their verbal and written consent before participation. All procedures with volunteers were conducted according to the Declaration of Helsinki.

Healthy men or women volunteers without neck or shoulder pain in the last three months were included. Subjects were excluded if they had previous surgery on the neck or shoulder, neuromuscular disorders, degenerative illness, neck or shoulder pain, or professional sports activities. A convenience sample was collected from the Clinica Centro Sur Medical Centre in Ciempozuelos (Madrid, Spain) and the University of Alcalá Campus in Alcalá de Henares (Madrid, Spain) through announcements.

A sample size of 20 subjects was selected based on the calculations of Kilem Li Gwet, who estimates that a sample size of 20 subjects with two raters, two ratings per rater, and an estimated ICC value of 0.90, leads to a 95% confidence interval (CI) length of 0.15 [17].

### 2.2. Examiners

Two muscle skeletal sonographers with at least 8 years of clinical experience performed the test by taking two measurements each on each side in a paired manner.

### 2.3. Procedure Assessment

**Figure 1:**
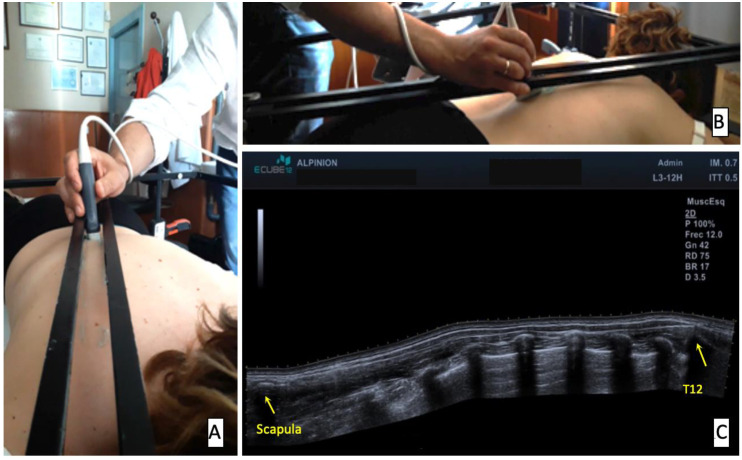
Volunteer setup and panoramic image using SIG_VIP for sampling (**A**,**B**) and the obtained image (**C**). Yellow arrows refer to the anatomical landmarks of spine of the scapula and spinous process of T12, origin, and insertion of lower fibers of the trapezius muscle and where the calipers were located to measure the length.

For the measurement of the length of the right and left lower trapezius muscles, an Alpinion ECube12 ultrasound device (Alpinion Medical Systems Co., Ltd., Seoul, Korea) was used with an L3-12H linear array, a 64 mm footprint, and a panoramic view active license. The panoramic view requires a straight line to have good vision and no artefacts. The muscle to be studied has to be located near the surface, and the movement of the scapula could influence the measurements if any pressure is placed on this region or the probe. A customized support [15] (SIG_VIP^®^, Pozuelo de Alarcon, Spain) was used and attached to a splint, which the patient laid under, in a prone position with the face supported by a facial hole. The arms were held at 90° abduction and the feet rested on a pillow. Surface anatomical landmarks (T12 spinous process and the spinae of scapula) were localized by one of the researchers manually and confirmed by an ultrasound assessment, and marked with a pen on the skin. Finally, the SIG_VIP^®^ was adjusted to line up the anatomical landmarks confirmed by the ultrasound (Figure 1).

This rail was fully covered with a transductor gel to ensure contact while moving the probe during the panoramic view acquisition (see Video S1) and to avoid pressure. Images were exported from the sonograph, and post-acquisition analysis was performed using the free software ImageJ [18] to measure the images, which was undertaken by each rater in their own studies.

### 2.4. Statistical Analysis

For the descriptive analysis of quantitative variables, the mean and standard deviation (SD) were used. For categorical variables, absolute frequencies and percentages were used. The outcome measures of this study were the length of the right and left lower trapezius muscles and the intra- and inter-rater reliability of their measurements on both sides with the SIG_VIP^®^. For the intra-rater reliability analysis, the ICC was calculated under the assumption of a two-way mixed model with absolute agreement and a single score (ICC_3,1_). For the inter-rater reliability analysis, the ICC was calculated under the assumption of a two-way mixed model with absolute agreement and an average score (ICC_3,2_).

The standard error of measurement (SEM) was calculated based on the mean squared error (MSE) of the repeated measures analysis of variance (ANOVA) using the formula √MSE. The minimal detectable change (MDC) with 95% confidence intervals was calculated using the formula SEM × √2 × 1.96. The SEM and MDC are also reported as a percentage of the sample mean (%SEM and %MDC). The presence of systematic error was evaluated with a repeated-measures ANOVA [19].

Bland-Altman plots were also constructed, and the relationship between the error of measurement and the mean value was evaluated with the Pearson correlation coefficient (r). It was considered that there was a relationship if the correlation coefficient was statistically significant. The mean differences (x) and limits of agreement (LoA) are also reported [20].

All analyses were performed with the statistical soft software R version 3.5.3 (R Core Team (2019), R Foundation for Statistical Computing, Vienna, Austria). All analyses were conducted while considering an α level of 0.05 as significant with 95% confidence intervals (CI).

## 3. Results

The sample was composed of 20 volunteers (11 female) with a mean age of 37.25 (SD, 12.90; range, 20–56) years. The demographic characteristics of the volunteers are presented in Table 1.

### Reliability of Length Measurements of Inferior Fibres of Trapezius Muscle

The descriptive statistics of the measurements and the within-rater differences are presented in Table 2. The repeated-measures ANOVA revealed no systematic errors between measurements (all *p* > 0.05). There was very good intra-rater reliability for sonographer 1 for the right side (ICC_3,1_ = 0.94; 95% CI, 0.86 to 0.98) and left side (ICC_3,1_ = 0.94; 95% CI, 0.85 to 0.97). There was also very good intra-rater reliability for sonographer 2 for the right side (ICC_3,1_ = 0.96; 95% CI, 0.90 to 0.98) and left side (ICC_3,1_ = 0.92; 95% CI, 0.80 to 0.97). Finally, there was good inter-rater reliability for the right side (ICC_3,2_ = 0.89; 95% CI, 0.72 a 0.96) and left side (ICC_3,2_ = 0.84; 95% CI, 0.61 a 0.94). The values of SEM and MDC are presented in Table 3.

**Figure 2:**
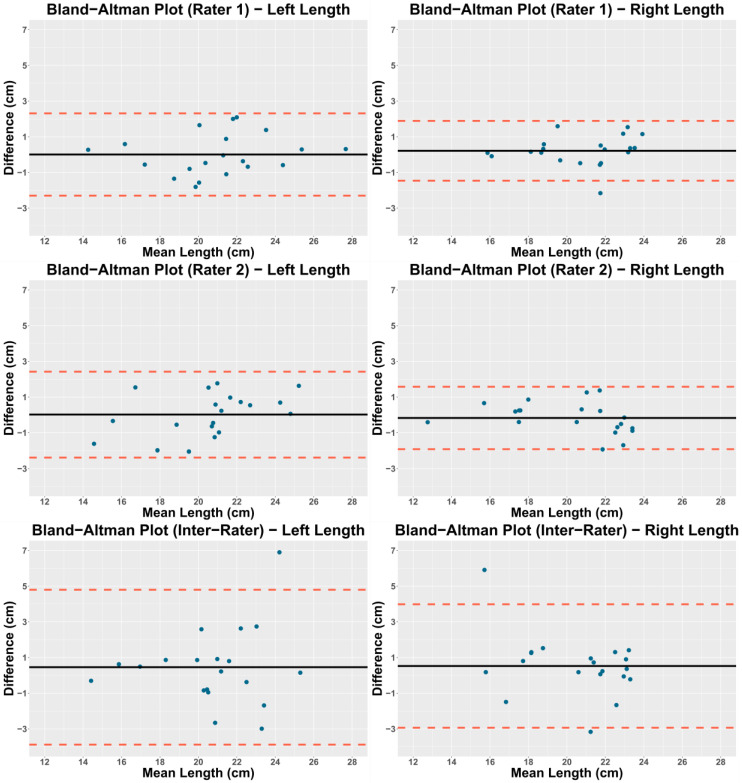
Bland-Altman plots.

The mean difference values between measurements for the intra- and inter-rater reliability analyses were close to zero (x = −0.17 to 0.52), indicating an absence of a systematic error. There were no statistically significant correlations between differences and mean values (all *p* > 0.05) (Table 4). The Bland-Altman plots are presented in Figure 2.

## 4. Discussion

The main findings of the study showed high rates of reliability and acceptable minimal detectable changes in the longitudinal axis. Some authors have suggested using the mean of three measurements to calculate the ICC, but we used only two measurements because of similar results [21]. The study of muscle morphology using sonography began with cross-sectional areas in different anatomical regions, and these studies examined clinical applications, such as lumbar spine or pelvic floor measurements [4,22], with applications in research and clinical settings for screening. This type of sonography has shown morphological changes that correlate with pain in subjects under experimental pain [23], under microgravity [24], with recurrent low back pain [4], or with shoulder pain [25].

The gold standard for morphological measurements is the MRI. Morphological measurements can be taken in the longitudinal axis or the transversal axis. In the transversal axis, observational studies [26,27] and a systematic review [28] have shown that MR has a high correlation between morphological changes and pathology or pain. However, to the best of our knowledge, there are no studies of longitudinal axis evaluation using MRI. This is surprising because this is the axis on which the muscle works.

Sonography in the longitudinal axis is performed using panoramic views. Nevertheless, to the best of our knowledge, panoramic views in longitudinal axis measurements have only been conducted for tendons [10,11,29]. The study of Ryan et al. [11] was the first to examine the test–retest reliability of sonography on the longitudinal axis for the Achilles tendon region. They found a high level of confidence (ICC = 0.95), and the MDC and SEM found were also similar to those of our results. In addition, Barfod et al. [10,29] found a high level of confidence (ICC = 0.96). Whereas, Ryan et al. used an approach with minimal support to guide the probe, Barfod et al. used a hands-free method without any guiding system to move in a straight line, thus showing that a guide is not necessary to do the measurement. These findings could be because these authors [10,11,29] used the panoramic view on the longitudinal axis to evaluate hard tissue, such as the Achilles tendon, rather than soft tissue, such as a muscle. Furthermore, Barford et al. advised that a soft tissue evaluation should require consideration of the deformation applied by the sonographer.

Previous validation studies of panoramic view ultrasound compared with MRI [7,8,30] are important for supporting the present study. However, although these studies used a panoramic view, they assessed the cross-sectional area rather than the longitudinal axis. This is a key difference because assessment in the longitudinal axis for long muscles requires a greater displacement of the probe and, as a result, attention is divided between the screen and the probe. Therefore, our study was designed to examine the reliability of a low-cost and quick method to perform measurements in the longitudinal axis, decreasing the need to split attention between the screen and the probe.

Finally, the decrease of activation of the inferior fibers of the trapezius muscle has been proposed as a contributor to scapular dyskinesis. In addition, it has been hypothesized that this decrease of activation produces morphological changes related to neck pain and shoulder pain. Numerous studies [31,32,33] have shown that a specific exercise program of activation of scapulothoracic muscles, such as the lower trapezius, benefits patients with neck or shoulder pain. Nevertheless, it is unknown whether these results are a consequence of the analgesic effect of physical exercise itself, or of the morphological changes occurring as a consequence of the muscle’s activation. Addressing this issue could help clinicians to choose the appropriate exercise. Thus, our results could be a preliminary step towards an inexpensive and flexible method to assess the impact of specific exercise treatments on the population with neck or shoulder pain.

We acknowledge some limitations of this study. The sample size is relatively small. Although there is no consensus on sample size calculations for reliability studies using ICC, some authors recommend more than 20 subjects for two raters and two measurements per rater [17,34]. The small sample size could have limited the power of the study to accurately detect good inter-rater reliability. Therefore, future studies should be conducted with greater sample sizes. In addition, the results from this study should be compared with other patient studies. Our study also has strengths. In particular, this was the first panoramic ultrasound study to validate the longitudinal axis assessment for muscles in the spine area. Offering the possibility of assessing larger muscles in the longitudinal axis, since it has been shown in muscles such as lower trapezius and its association with musculoskeletal disorders. In addition, it is a low-cost, flexible, and reliable morphological evaluation method. Thus, the use of SIG_VIP^®^ facilitates the sampling of large panoramic ultrasounds, thereby enabling this work in clinical practice. In this study, this tool demonstrated high precision, allowing reliable image acquisition with attention focused on the screen. However, although the use of SIG_VIP^®^ only requires 1′43″, this time is significantly longer than without any support.

## 5. Conclusions

To the best of our knowledge, this is the first panoramic ultrasound study to validate the longitudinal axis assessment for muscles in the spine area. Our results showed that the SIG_VIP^®^ has high reliability for panoramic views of the inferior fibers of the trapezius in the longitudinal axis, with good intra- and inter-rater reliability for right and left lower trapezius. Therefore, we defined a novel low-cost, flexible, and reliable approach that allows reliable image acquisition with attention focused on the screen, facilitating large panoramic ultrasounds evaluations for clinical and research purposes.

## Figures and Tables

**Table 1:** Demographic characteristics of volunteers (*n* = 20).

Variable	Mean (SD)	Range
Age, years	37.25 (12.90)	20–56
Weight, kg	68.74 (15.41)	46–100
Height, cm	170.55 (11.35)	150–186
BMI, kg/m^2^	23.42 (3.62)	18.96–30.86
Sex, *n* (%)		
Male	9 (45)	-
Female	11 (55)	-
Dominant side, *n* (%)		
Right	20 (100)	-
Left	0 (0)	-
Smoker, *n* (%)	5 (25)	-
Worker, *n* (%)	15 (75)	-
Sports Practice, *n* (%)	13 (65)	-

Abbreviations: SD: Standard deviation; BMI: Body mass index.

**Table 2:** Descriptive statistics of lower trapezius muscle measurements.

Variable	First Measurement Mean (SD)	Second Measurement Mean (SD)	Mean Measurement * Mean (SD)	Difference ** Mean (SD)
Right Length, cm				
Rater 1	20.86 (2.54)	20.65 (2.42)	20.76 (2.44)	0.21 (0.84)
Rater 2	20.14 (2.89)	20.31 (3.20)	20.23 (3.01)	−0.17 (0.87)
Left Length, cm				
Rater 1	21.00 (3.23)	20.99 (3.05)	21.00 (3.09)	0.01 (1.15)
Rater 2	20.55 (3.11)	20.53 (2.60)	20.54 (2.81)	0.02 (1.20)

Abbreviations: SD: Standard deviation. * Mean measurement refers to the mean of the two measurements of each rater. ** Difference refers to the mean difference between the two measurements of each rater.

**Table 3:** Reliability of lower trapezius muscle measurements.

Rater	ICC (95% CI)	ANOVA *p*-Value	SEM, cm	%SEM	MDC, cm	%MDC
Right Length						
Rater 1 (ICC_3,1_)	0.94 (0.86, 0.98)	0.27	0.59	2.86	1.64	7.92
Rater 2 (ICC_3,1_)	0.96 (0.90, 0.98)	0.39	0.62	3.06	1.71	8.48
Inter-rater (ICC_3,2_)	0.89 (0.72, 0.96)	0.19	1.22	5.97	3.39	16.55
Left Length						
Rater 1 (ICC_3,1_)	0.94 (0.85, 0.97)	0.98	0.82	3.88	2.26	10.76
Rater 2 (ICC_3,1_)	0.92 (0.80, 0.97)	0.94	0.85	4.13	2.35	11.46
Inter-rater (ICC_3,2_)	0.84 (0.61, 0.94)	0.36	1.53	7.39	4.25	20.48

Abbreviations: ICC: Intraclass correlation coefficient; CI: Confidence intervals; ANOVA: Analysis of variance; SEM: Standard error of the measurement; MDC: Minimal detectable change.

**Table 4:** Bland-Altman plot statistics.

Rater	X (cm)	LoA (cm)	r	*p*-Value
Right Length				
Rater 1	0.21	−1.46 to 1.89	0.15	0.53
Rater 2	−0.17	−1.92 to 1.58	−0.36	0.12
Inter-rater	0.52	−2.94 to 3.99	−0.35	0.14
Left Length				
Rater 1	0.01	−2.30 to 2.31	0.16	0.50
Rater 2	0.02	−2.38 to 2.42	0.43	0.06
Inter-rater	0.46	−3.88 to 4.80	0.14	0.56

Abbreviations: x: Mean difference; LoA: Limits of agreement; r: Pearson correlation coefficient.

## Supplementary Materials

The following are available online at https://www.mdpi.com/1660-4601/17/19/7123/s1. Video S1: How SIG_VIP^®^ works.
